# Vascularite cutanée paranéoplasique révélant un lymphome non hodgkinien

**DOI:** 10.11604/pamj.2014.17.134.3994

**Published:** 2014-02-26

**Authors:** Wafa Chebbi, Baha Zantour

**Affiliations:** 1 Service de Médecine Interne, CHU Taher Sfar Mahdia, 5100 Mahdia, Tunisie

**Keywords:** Vascularite cutanée, lymphome non-hodgkinien, hémopathie maligne

## Image en medicine

La vascularite cutanée paranéoplasique est une entité rare observée principalement au cours des syndromes myélodysplasiques, des leucémies à tricholeucocytes et beaucoup plus rarement au cours des lymphomes. Le tableau clinique est très polymorphe: purpura vasculaire ou placards ecchymotiques, lésions nodulaires, vésicobulleuses, ulcérations voire nécroses cutanées. Nous rapportons l'observation d'une patiente âgée de 42 ans, qui consultait pour des plaques ecchymotiques, infiltrées, douloureuses et nécrotiques par endroits, siégeant au niveau des jambes et associées à des arthralgies inflammatoires des chevilles. L'examen clinique ne révélait aucune anomalie, à l'exception des lésions cutanées. Il y avait un syndrome inflammatoire biologique. La numération formule sanguine, le bilan rénal et hépatique étaient normaux. La radiographie thoracique et l’échographie cardiaque était normales. Le bilan immunologique (AAN, ANCA, cryoglobuline, facteurs rhumatoïdes, anticorps anti-phospholipides) était négatif. La biopsie cutanée montrait une vascularite leucocytoclasique avec immunofluorescence directe négative. Une corticothérapie à la dose de 0,5 mg/Kg/j était instaurée avec disparition des arthralgies et des lésions cutanées. Trois mois plus tard, la patiente était réhospitalisée pour une récidive de la vascularite cutanée associée à une fièvre à 39°C, une pâleur cutanéo-muqueuse et des adénopathies cervicales et axillaires. Le bilan biologique montrait un syndrome inflammatoire et une anémie à 8g/dl d'hémoglobine. Les hémocultures étaient négatives. La biopsie ganglionnaire montrait une localisation d'un lymphome malin non hodgkinien de phénotype B. L'institution d'une chimiothérapie a permis d'obtenir une disparition complète et rapide des lésions cutanées. La recherche des signes cliniques et biologiques d'une hémopathie maligne et en particulier d'un lymphome doit figurer dans le bilan étiologique d'une vascularite cutanée.

**Figure 1:**
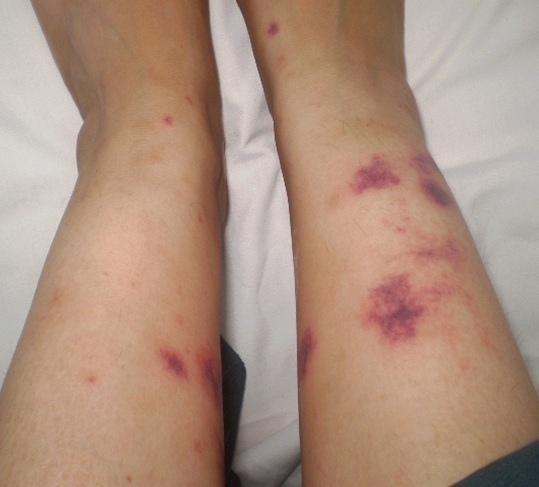
Plaques ecchymotiques et nécrotiques des jambes

